# Integrated analysis of competitive endogenous ribose nucleic acids (ceRNAs)-related regulatory networks in invasive and non-invasive non-functioning pituitary adenomas (NFPAs)

**DOI:** 10.3389/fsurg.2022.983958

**Published:** 2022-09-29

**Authors:** Jiangtao Liu, Kaixuan Wang, Hongming Ji, Gangli Zhang, Shengli Chen, Shiyuan Zhang, Fake Lu, Changchen Hu

**Affiliations:** ^1^ Department of Neurosurgery, Shanxi Provincial People’s Hospital, Shanxi Medical University, Taiyuan, China; ^2^ Department of Biomedical Engineering, Binghamton University, State University of New York, Binghamton, United States; ^3^ Department of Neurosurgery, Shuozhou People’s Hospital, Shuozhou, China

**Keywords:** pituitary adenomas, competitive endogenous RNA, regulatory networks, nonfunctional adenoma, invasiveness, non-coding RNA

## Abstract

**Background:**

This study aims to identify the differentially expressed (DE) non-coding ribose nucleic acids (ncRNAs), messenger RNA (mRNA) expression profiles, and competitive endogenous RNA (ceRNA)-related regulatory networks in invasive and non-invasive nonfunctioning pituitary adenomas (NFPAs).

**Methods:**

A full-transcriptome sequencing of invasive and non-invasive NFPAs is carried out to evaluate the expression profiles of circular RNAs (circRNAs), long non-coding RNAs (lncRNAs), microRNAs (miRNAs), and mRNA expression profiles.

**Results:**

The screening criteria resulted in 118 DEcircRNAs (88 up-regulated and 30 down-regulated), 105 DElncRNAs (68 up-regulated and 37 down-regulated), 43 DEmiRNAs (22 up-regulated and 21 down-regulated), and 268 DEmRNAs (194 up-regulated and 74 down-regulated). Accordingly, a ceRNA regulatory network related to invasive NFPA is constructed. Further, the Gene Ontology and Kyoto Encylopedia of Genes and Genomes analyses showed that circRNAs and lncRNAs in the network are related to chromatin remodeling, participating in the Janus kinase/signal transducer and activator of transcription (JAK-STAT) and calcium signaling pathways. Hsa-miR-1248 showed exceptional connectivity in the ceRNA regulatory network, which could be closely related to the invasiveness of NFPAs.

**Conclusions:**

Together, these findings clarified the regulatory mechanisms of invasive and non-invasive NFPAs, providing innovative research avenues and therapeutic targets for invasive NFPAs.

## Background

Pituitary adenoma (PA) is one of the most common benign intracranial tumors on the pituitary gland. This rare pathological condition often results from excessive secretion of growth hormone. The PA condition can be classified into functioning pituitary adenomas (FPAs) with characteristic symptoms of acromegaly or Cushing’s disease and non-functioning pituitary adenomas (NFPAs). The NFPAs are usually caused by gonadotropin-secreting cells, resulting in the reduced secretion of pituitary hormone. Moreover, NFPAs can be accessibly diagnosed at a specific tumor size as it begins to oppress the intracranial nerve and surrounding brain tissues. Although most PAs are biologically benign, some PAs can migrate to the important structures around the cavernous sinus, internal carotid artery, and optic chiasma. In rare cases, PAs are accompanied by severe metastasis, leading to challenges in their complete surgical resection and a high risk of recurrences even being removed entirely. Hitherto, the precise mechanism related to the invasiveness of NFPA is yet to be explored, requiring the discovery of various vital biomarkers. In addition, it is required to investigate specific genetic changes to explore invasive NFPA further and provide evidence to support the molecular theranostics of the disease.

In recent times, tremendous advancements have resulted in the development of high-throughput sequencing technology to investigate the expression profiles of non-coding ribose nucleic acids (ncRNAs), including circular RNA (circRNA), long non-coding RNA (lncRNA), and micro RNA (miRNA). Initially, these ncRNAs were regarded as “junk molecules” regarding gene expression, which have been further identified as biomarkers for disease prognosis and regulators of various cellular processes. In this context, several transcriptome studies confirmed a significant correlation between ncRNA and human diseases, including oncological, neurological, and developmental diseases (1, 2). Recently, a study indicated that different RNAs could interact and regulate the expression of genes at the post-transcriptional level through complex molecular mechanisms, in which competitive endogenous RNA (ceRNA) networks played essential roles in various physiological processes (3). CeRNAs, a general term representing different transcripts, possess similar miRNA response elements (MREs) and constitute a competitive relationship, including circRNAs, lncRNAs, mRNAs, and pseudogenes. Notably, ceRNAs bind to similar miRNAs, forming a complex RNA regulatory network. These RNA regulatory networks not only regulate the ceRNA expression levels but also influence each other, eventually affecting the biological process of cells. Consequently, a potential ceRNA network, namely the lncRNA-circRNA-miRNA-mRNA network, can be established based on the same miRNAs. For instance, the integrated analysis of the ceRNA network confirmed that the ceRNA network played an important role in the progression of brain tumors (4, 5).

Despite the success in establishing the ceRNA network, the characteristic comprehensive expression profiles of circRNAs, lncRNAs, miRNAs, and mRNAs related to invasive NFPA, as well as the mechanism of ceRNA-related regulatory networks, remain unclear. Motivated by these considerations, this study aims to construct a gene regulatory network model of invasive NFPA by ceRNAs. Further, we analyze and provide new ideas and methods for further understanding the disease. Finally, the regulatory mechanisms of invasive and non-invasive NFPAs are clarified, providing innovative research avenues and therapeutic targets for invasive NFPAs.

## Materials and methods

### Patients and tissue collection

**Figure 1:**
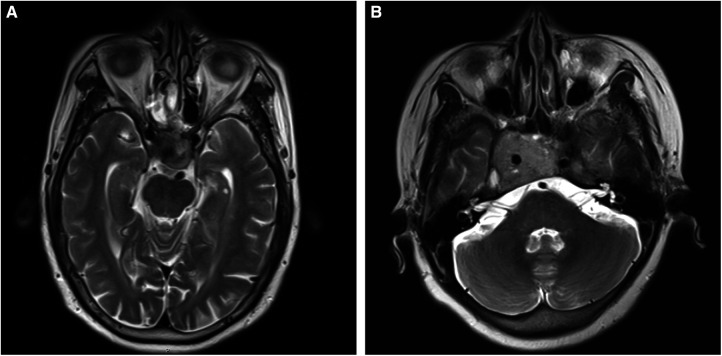
(**A,B**) T2-weighted images of nonfunctional pituitary adenomas with Knosp grades 3 and 4, respectively.

The tissue samples were collected from the selected patients with invasive and non-invasive NPFA (*n* = 6 each) who underwent transsphenoidal surgery at the Shanxi Provincial People’s Hospital, Shanxi, PR China, from 2020 to 2021. It should be noted that the patients had not received radiotherapy or chemotherapy before surgery. Further, the diagnosis of NFPA was confirmed by the histopathological analysis. The tumor invasion was defined as Knosp grade 3 or 4 (Figure 1), or sphenoid sinus invasion was detected during surgery. The resected tumor specimens were preserved in liquid nitrogen for 2 h and then stored in a freezer at −80°C. Further, the high-throughput sequencing was performed to analyze ncRNA and mRNA expression profiles in all invasive and non-invasive NPFA (*n* = 12) samples and validated by quantitative reverse transcription polymerase chain reaction (qRT-PCR) analysis. The experimental protocols were approved by the ethical committee of the Shanxi Provincial People’s Hospital. The informed consent was obtained from all patients.

### Library building and sequencing

Initially, the total RNA extracted from tissues or cells was purified using 1% agarose gel electrophoresis to detect RNA degradation and contamination. Then, the preliminary NanoPhotometer® spectrophotometer (IMPLEN, Westlake Village, CA, USA) was employed to check the RNA purity. Further, the Qubit® RNA Assay Kit in Qubit® 2.0 Fluorometer (Life Technologies, Carlsbad, CA, USA) was applied to quantify the RNA concentration. Finally, the RNA Nano 6000 Assay Kit of the Agilent Bioanalyzer 2,100 system (Agilent Technologies, CA, USA) was utilized to confirm RNA integrity.

The miRNA library was initially constructed using NEBNext® Multiplex Small RNA Library Prep Set for Illumina® (NEB, USA). Further, lncRNA, mRNA, and circRNA libraries were fabricated by NEBNext® Ultra™ Directional RNA Library Prep Kit for Illumina® (NEB, USA) (6). The fabricated libraries were preliminarily quantified using Qubit2.0, and the insert size of the library was then detected using an Agilent 2,100 Bioanalyzer. After the insert size met the expectations, the effective concentration of the library was then quantified precisely by the qRT-PCR analysis to substantially ensure the quality of RNA libraries. Further, the sequencing was executed using an IlluminaHiSeq™2500/MiSeq Illumina Hiseq platform, based on the effective concentration of the RNA library and the demand for data output pooling. In this study, the library construction and RNA sequencing were accomplished with the support of Nuohe Zhiyuan Technology Co., Ltd. (Beijing, China).

### Data filtering

Typically, the original sequencing data often contain a small number of reads with sequencing connectors or low sequencing quality. To overcome this aspect, the sequencing data were filtered to ensure the quality and reliability of the data analysis. In the cases of lncRNA, circRNA, and mRNA, the purified data (clean reads) were obtained by eliminating the reads containing adapter, reads containing ploy-N, and low-quality reads from raw data. For miRNA, the raw data were purified by removing reads containing ploy-N, with 5′ adapter contaminants, without 3′ adapter or the insert tag, containing ploy A or T or G or C, and low-quality reads. Simultaneously, Q20, Q30, and GC content of the clean data were calculated. It should be noted that all the downstream analyses were based on clean data with high quality.

### Data analysis

The differentially expressed (DE) ncRNAs (including DElncRNAs, DEcircRNAs, and DEmiRNAs), as well as DEmRNAs, were screened using the DESeq2 R package (1.8.3) (7). The default threshold for differential expression, i.e., the adjusted *p*-value (*p*_adj_), was obtained by the multiple test correction of *p*-values using the Benjamini-Hochberg method at the high false positive rate. Notably, a smaller *p*_adj_ value would indicate a more significant result. Therefore, *p*_adj_ < 0.05 was considered a defined level of statistically significant difference. If *p*_adj_ < 0.05 with no difference, *p* < 0.05 was then used for differential screening in subsequent analysis. Further, Ggplot2 and pheatmap packages were applied to draw volcano and heat maps of the expression profiles of DElncRNAs, DEmiRNAs, and DEmRNAs, respectively.

### Construction of miRNA regulatory network

According to the DEmiRNA target genes (mRNA), miRNA target genes were analyzed to predict the intersection of miRanda and RNAhybrid softwares (8–10). Further, the target mRNAs corresponding to DEmiRNAs and mRNAs-overlapped DEmRNAs were analyzed. Considering the inhibitory effect of miRNAs on mRNAs, the combinations of significantly down-regulated miRNAs, and significantly up-regulated mRNAs, as well as significantly up-regulated miRNAs, and significantly down-regulated mRNAs were selected as target gene pairs to generate the miRNA-mRNA regulatory network.

### Construction of ceRNA regulatory network

Notably, lncRNAs and circRNAs possess several miRNA-binding sites that can act as miRNA sponges to competitively constrain the regulatory effects of miRNAs on their target genes, thus indirectly regulating gene expression. According to the ceRNA theory, circRNA/lncRNA target gene pairs were identified with the same miRNA-binding sites. Then, the circRNA-miRNA-mRNA regulatory relationship was constructed with lncRNAs and circRNAs as decoys, miRNA as the core, as well as mRNAs as the target to create a ceRNA regulatory network.

### Functional analyses

The Gene Ontology (GO) and Kyoto Encyclopedia of Genes and Genomes (KEGG) pathway analyses were carried out based on the results of the screened target gene (mRNA). In addition, the functions of lncRNAs and circRNAs were predicted using the Cytoscape software (version 6.7) to predict mRNAs in the invasive NFPA-related ceRNA networks (11).

## Results

### Differential expression analysis

**Figure 2:**
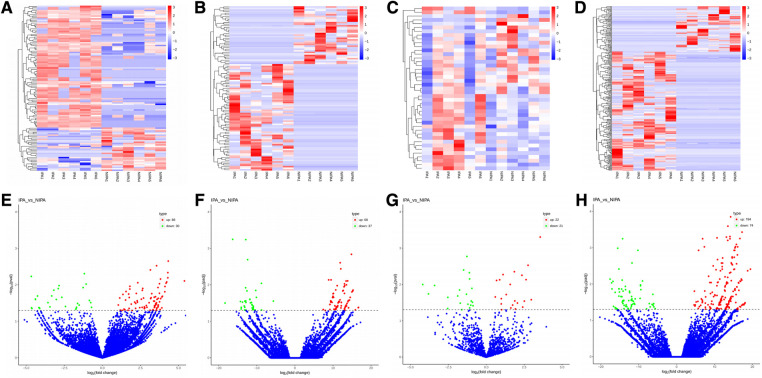
(**A**–**D**) Cluster heat map of DEcircRNAs, DElncRNAs, DEmiRNAs, and DEmRNAs. Red indicates up-regulation and blue indicates down-regulation. (**E–H**) Volcano map of DEcircRNAs, DElncRNAs, DEmiRNAs, and DEmRNAs. Red indicates up-regulation and green indicates down-regulation. DE, differential expression; circRNA, circular RNA; lncRNA, long chain non-coding RNA; IPA, invasive nonfunctional pituitary adenomas; NIPA, non-invasive nonfunctional pituitary adenomas.

**Figure 3:**
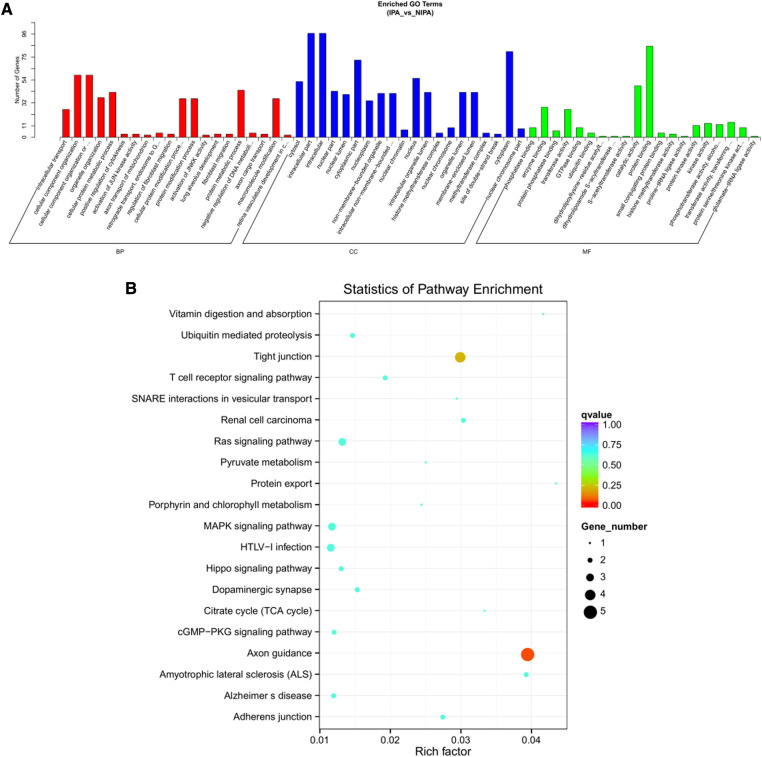
DEcircRNA target gene enrichment analysis. (**A**) GO function enrichment bar chart of the top 20 enriched items. Abscissa denotes GO entry: BP, biological process; CC, cellular component; MF, molecular function represented by different color bars; ordinate is the number of genes enriched by GO entry. (**B**) KEGG pathway enrichment bubble diagram.

**Figure 4:**
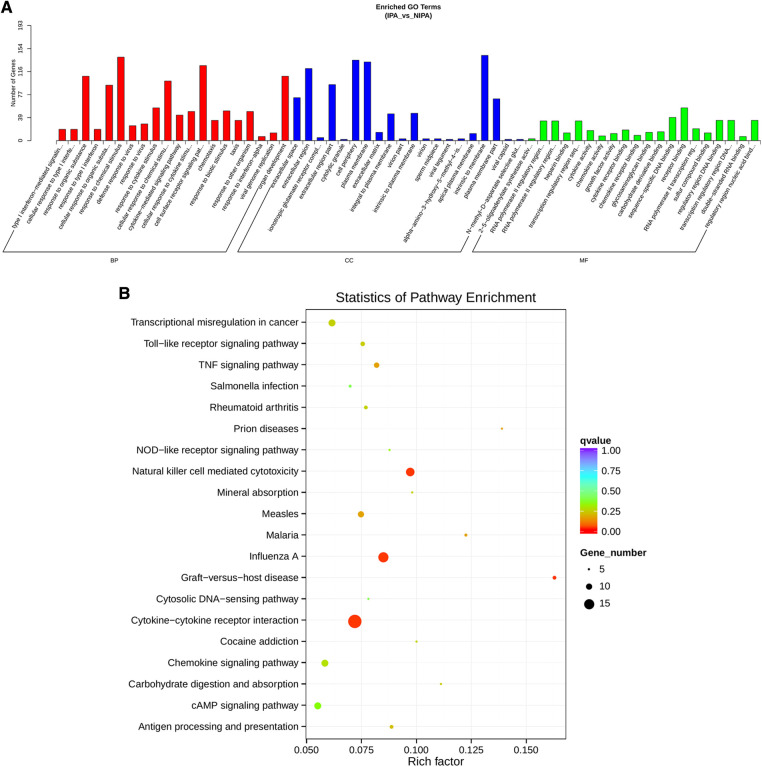
DElncRNA target gene GO and KEGG enrichment analyses. (**A**) Top 20 GO-BP, CC, and MF entries. BP, biological process; CC, cell components; MF, molecular function. (**B**) Top 20 enrichment pathways.

Based on the screening criteria, various RNAs and their altered expressions were identified, such as 118 DEcircRNAs (88 up-regulated and 30 down-regulated), 105 DElncRNAs (68 up-regulated and 37 down-regulated), 43 DEmiRNAs (22 up-regulated and 21 down-regulated), as well as 268 DEmRNAs (194 up-regulated and 74 down-regulated). Figure 2 shows the distribution of DElncRNAs, DElncRNAs, and DEmRNAs as cluster heat maps and volcano maps. It was observed from the results that the invasive and non-invasive NFPA could be significantly separated, indicating reliable differential expression analysis. Further, the enrichment of DEcircRNAs and DElncRNAs by GO and KEGG analyses were analyzed, representing that DEcircRNAs were significantly related to the cellular composition, tissue or biogenesis, cellular protein metabolism, and tight junctions, as well as axonal orientation (Figure 3). In this context, DElncRNAs were substantially associated with the cell surface receptor signaling pathways, cell responses to chemical stimuli, and cytokine–cytokine receptor interactions (Figure 4).

### Construction of miRNA regulatory network

**Figure 5:**
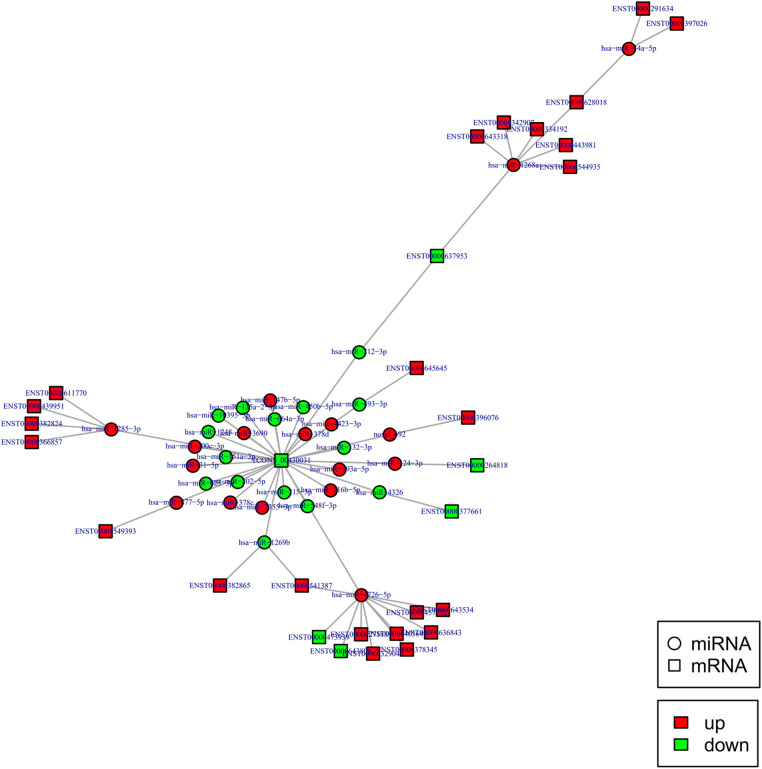
DEmiRNA and targeted DEmRNA regulatory network. Circle: miRNA, square: lncRNA. Red: up-regulation, green: down-regulation.

**Figure 6:**
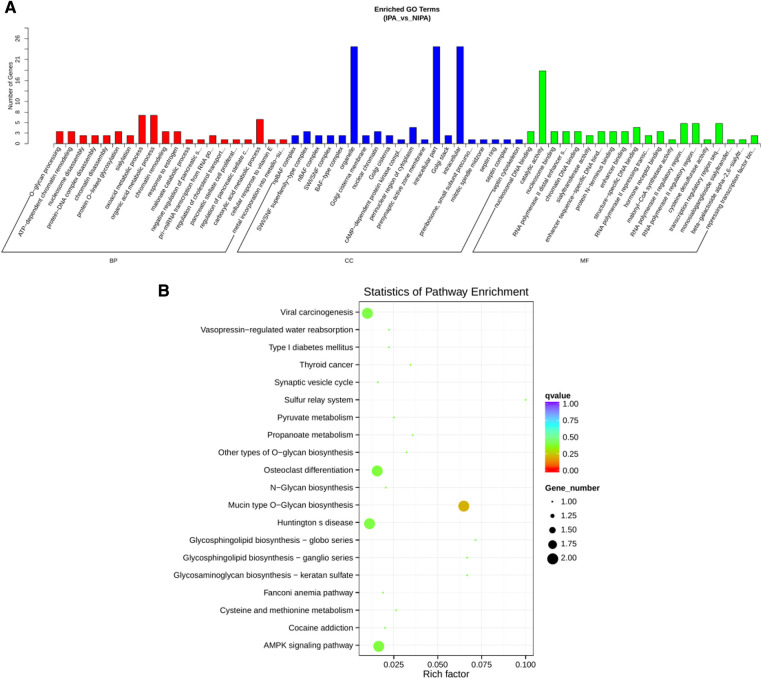
miRNA regulatory network target gene enrichment analysis. (**A**) GO function enrichment bar chart of the top 20 enriched items. Abscissa denotes GO entry, BP, biological process; CC, cellular component; MF, molecular function are represented by different color bars; ordinate is the number of genes enriched by GO entry. (**B**) KEGG pathway enrichment bubble diagram of top 20 enriched pathway entries.

To construct the miRNA regulatory network, the combinations of differentially expressed miRNAs and mRNAs were identified, in which 15 significantly down-regulated miRNAs, and 24 significantly up-regulated mRNAs, as well as 17 significantly up-regulated miRNAs, and 6 significantly down-regulated mRNAs were selected as target gene pairs. These selected target genes were then entered into Cytoscape to obtain a total of 62 sides of a DEmiRNA–DEmRNA regulatory network (Figure 5). Further, the target genes corresponding to the DEmiRNA–DEmRNA regulatory network were examined by the GO functional enrichment (Figures 2, 3) and KEGG enrichment analyses (Figure 6). On the one hand, the GO functional enrichment analysis showed that the target genes of DEmiRNAs were substantially related to the sequence-specific deoxyribonucleic acid (DNA) binding of the transcriptional regulatory region, sequence-specific DNA binding of the RNA polymerase II regulatory region, and structure-specific DNA binding. On the other hand, the KEGG pathway analysis showed that DEmRNAs might be involved in the O-glycan synthesis, adenosine 5′-monophosphate (AMP)-activated protein kinase (AMPK) signaling pathway, osteoclast differentiation, and viral carcinogenesis.

### Construction of the circRNA-lncRNA coregulatory ceRNA network

**Figure 7:**
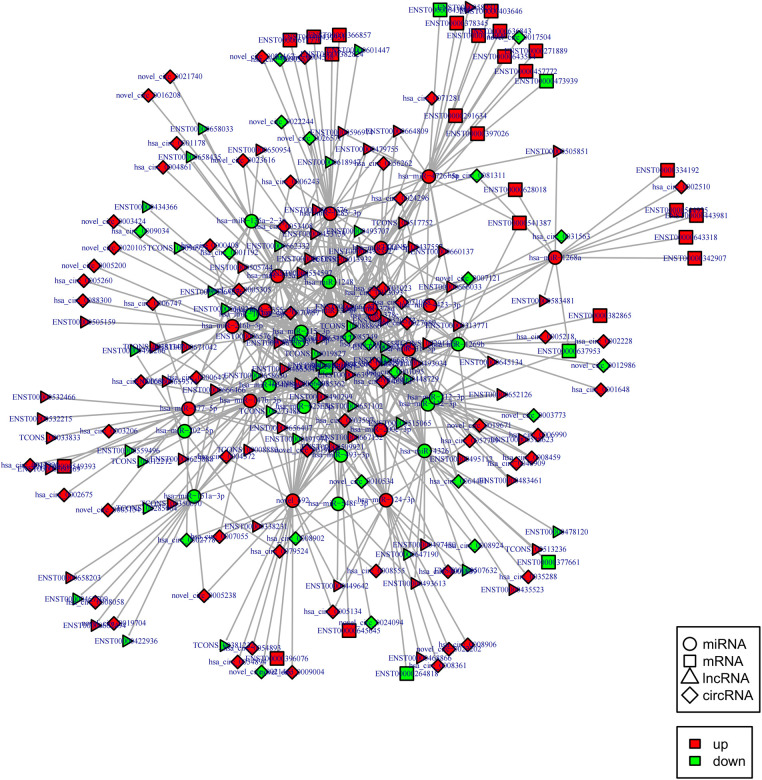
CircRNA-lncRNA co-regulated ceRNA network. Circle: miRNA, square: mRNAs, triangle: lncRNA, diamond: circRNA. Red: up-regulation, green: down-regulation.

Indeed, circRNAs and lncRNAs can bind to MRE and participate in the regulation of post-transcriptional expression. Considering this aspect, a circRNA–lncRNA co-regulatory ceRNA network was constructed to explore whether circRNAs and lncRNAs could share the same miRNA-mRNA relationship pair (same competing MRE). This regulatory ceRNA network included 242 nodes (61 up-regulated and 22 down-regulated circRNAs, 62 up-regulated and 35 down-regulated lncRNAs) and 654 edges (Figure 7). The network subsequently revealed that most circRNAs and lncRNAs functioned by jointly regulating miRNAs. For instance, it was predicted that the circRNA, hsa_circ_0005558, and lncRNA, CCDC144NL-AS1 displayed the same target, i.e., hsa-miR-1268a, as ceRNA could up-regulate SWI/SNF related, matrix-associated, actin-dependent regulator of chromatin, subfamily e, member 1(SMARCE1) to mediate chromatin remodeling.

**Figure 8:**
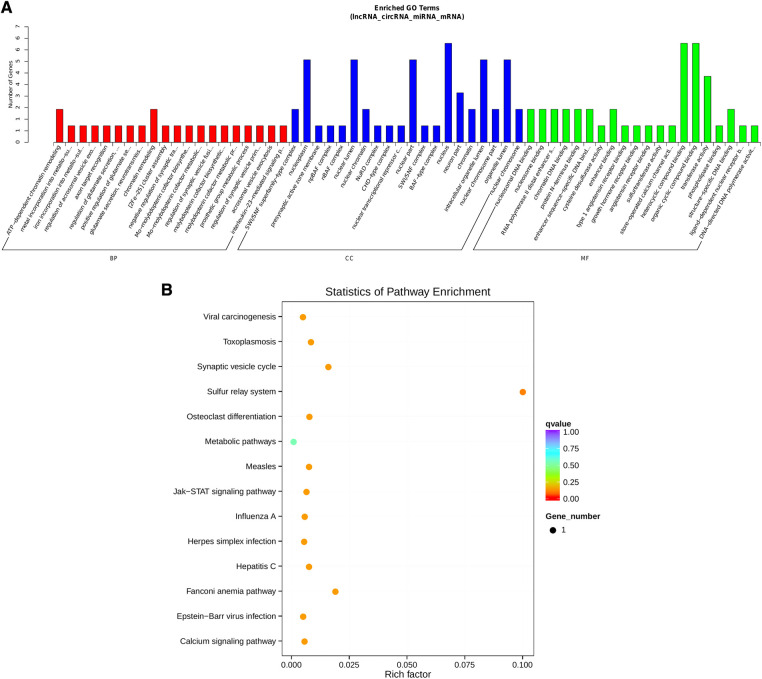
ceRNA regulatory network target gene enrichment analysis. (**A**) GO function enrichment bar chart of the top 20 enriched items. Abscissa denotes GO entry, BP, biological process; CC, cellular component; MF, molecular function represented by different color bars; ordinate is the number of genes enriched by GO entry. (**B**) KEGG pathway enrichment bubble diagram.

Notably, hsa-miR-1248 possessed the highest connectivity in the co-regulatory ceRNA network and the most number of node interactions, suggesting its potential role in invasive NFPA. To clarify the potential functions of lncRNAs and circRNAs in invasive NFPA, the GO and KEGG pathway enrichment analyses were further used to detect the critical functionalities of the abnormally expressed mRNAs in the lncRNA-circRNA co-regulated ceRNA network. It was observed that the lncRNAs and circRNAs in this regulatory network were mainly related to various biological processes, such as chromatin remodeling, closely related to tumor formation (Figure 7). In addition, the KEGG pathway analysis indicated that the Janus kinase/signal transducer and activator of transcription (JAK-STAT), as well as calcium signaling pathways, were enriched in the ceRNA regulatory network (Figure 8). Together, it could be concluded that these pathways involved in many intracellular metabolic processes could substantially play critical roles in the occurrence and development of invasive NFPA.

## Discussions

This study is aimed at identifying various DE ncRNAs and mRNA expression profiles, as well as ceRNA-related regulatory networks in invasive and non-invasive NFPAs. Accordingly, the full-transcriptome sequencing of invasive and non-invasive NFPA tissue samples resulted in various DE RNAs, including 118 DEcircRNAs (88 up-regulated and 30 down-regulated), 105 DElncRNAs (68 up-regulated and 37 down-regulated), 43 DEmiRNAs (22 up-regulated and 21 down-regulated), and 268 DEmRNAs (194 up-regulated and 74 down-regulated). Further, the GO and KEGG-based enrichment analyses of lncRNAs and circRNAs indicated that their target genes were associated with cell composition, tissue or biogenesis, cell protein metabolism, cell surface receptor signaling pathways, and cytokine-cytokine receptor interactions.

Considering the inhibitory effect of miRNAs on mRNAs, the abnormally expressed miRNAs and mRNAs were screened significantly to establish a cooperative regulatory network. The functional enrichment analysis of DEmRNAs in the miRNA regulatory network presented that the sequence-specific DNA binding of the transcriptional regulatory region, sequence-specific DNA binding of the RNA polymerase II regulatory region, and structure-specific DNA binding were closely associated with the invasive NFPA. In this context, previous studies indicated that the oncoprotein Ras instigated the transcriptional silencing of Fas and other tumor suppressor genes by substantially binding to ZFP354B (a sequence-specific DNA-binding protein) (12). In addition, several recent reports presented that the tumor suppressor p53 was a sequence-specific DNA-binding protein that significantly activated the gene transcription to regulate the survival and proliferation of cells (13). These results suggested that the role of mRNAs in growth hormone-secreting pituitary adenomas(GHPA) might be significantly related to the sequence-specific DNA binding. In addition, the KEGG pathway analysis displayed that O-glycan synthesis, AMPK signaling pathway, osteoclast differentiation, and viral carcinogenesis were the most abundant pathways of the target genes. Specifically, AMPK is a heterotrimeric protein composed of *α*, *β*, and *γ* subunits, which can be activated by cellular stress, increasing the AMP levels by allosteric binding of AMP to the *γ* subunit and phosphorylation of Thr172 in the h subunit by serine/threonine kinase 11, calcium/calmodulin-dependent protein kinase, and transforming growth factor-b activated kinase (14–16). Notably, in a case, it was demonstrated that inhibiting AMPK signaling could reduce gonadotropin secretion in NFPA (17). To this end, O-polysaccharides also play critical roles in the malignant progression of tumors, including promoting the migration, invasion, and metastasis of pancreatic cancer cells (18). Hence, mRNAs associated with invasive NFPA may play key roles through sequence-specific DNA binding and the AMPK signaling pathway.

Indeed, lncRNAs and circRNAs are considered molecular sponges of miRNAs due to the same miRNA-binding sites. In this regard, lncRNAs and circRNAs bind to the miRNAs and indirectly regulate the expressions of their downstream target genes through inhibition, representing a new relationship between ncRNAs and invasive NFPA. Therefore, a circRNA–lncRNA co-regulatory ceRNA network was constructed in this study. Specifically, miRNAs located between circRNAs/lncRNAs and mRNAs occupied the central position in the circRNA/lncRNA-miRNA-mRNA gene axis, indicating that these could significantly play essential roles in the treatment and diagnosis of diseases (19). In the present study, the down-regulated hsa-miR-1248 showed the highest degree of connectivity in the ceRNA regulatory network associated with invasive NFPA. Previous studies indicated that overexpression of thymidylate synthetase (TYMS) could suppress DNA synthesis and affect DNA methylation patterns, thus supporting cell proliferation, invasion, and tumor progression. In another case, it was demonstrated that hsa-miR-1248 bound to the 3′-untranslated region of the TYMS rs2790G allele and inhibited its expression in non-small cell lung cancer *in vitro* (20, 21), indirectly demonstrating a close relationship between hsa-miR-1248 and migration of tumors.

In this study, it was observed that hsa-miR-1285-3p, hsa-miR-4326, hsa-miR-4726-5p, hsa-miR-147b-5p, and hsa-miR-34a-5p showed high connectivity in the ceRNA regulatory network related to invasive NFPA. These miRNAs could regulate the expressions of various genes involved in many biological processes, including tumor cell proliferation, differentiation, migration, and invasion. Since the functionalities of these RNAs have been moderately confirmed, we believe that our results were in agreement with the findings of the previous studies indicating their potential roles in the invasive NFPA (22, 23). In this context, a report indicated that hsa-miR-1285-3p could directly inhibit the expression of the JUN oncogene in hepatocellular carcinoma (24). Further, it was validated and implied that hsa-miR-1285-3p could act as a potential tumor suppressor. In this study, the GO and KEGG analysis confirmed that the co-expressed mRNAs in circRNA-lncRNA co-regulated ceRNA network were closely related to the chromatin remodeling, as well as JAK-STAT signaling and calcium signaling pathways, involving in tumor cell proliferation, metastasis, invasion and immune regulation (25, 26). Together, these results confirmed that most circRNAs and lncRNAs in the ceRNA regulatory network were associated with tumor invasiveness.

Despite the success in constructing and analyzing the regulatory networks, the current study showcased some limitations in various aspects, as stated below. Firstly, the theoretical and experimental data for RNAs were insufficient, requiring further experiments to obtain more detailed information about the gene pathways and functions of the network. Secondly, the results were obtained from considerable a small sample size, requiring validation with larger sample size. Thirdly, more information about the mechanism of tumor invasion could be obtained by comparing NFPA with normal pituitary tissue. Finally, this study has just analyzed the invasiveness of NFPA with no exploration of details of tumor recurrence. Considerably, in addition to the above-notified investigations, addressing these limitations may provide new directions for further research.

## Conclusions

In summary, we have systematically analyzed the correlation between ncRNAs and invasive NFPA by full-transcriptome sequencing. In addition, a ceRNA regulatory network characterized by abnormally expressed circRNAs/lncRNAs/miRNAs and mRNAs were established. It was observed that various genes, including SMARCE1, chromodomain helicase DNA binding protein 4 (CHD4), tyrosine kinase 2 (TYK2), and calcium release-activated calcium modulator 2 (ORAI2), were involved in the chromatin remodeling, JAK-STAT signaling, and calcium signaling pathways. These pathways might substantially play important roles in the pathogenesis of invasive NFPA. Together, these findings validated the complexity of the genome networks in the invasive NFPA, suggesting potential new therapeutic targets for patients with invasive NFPA.

## Data Availability

The authors acknowledge that the data presented in this study must be deposited and made publicly available in an acceptable repository, prior to publication. Frontiers cannot accept a manuscript that does not adhere to our open data policies.
